# New diagnostic test for high risk of breast cancer in Poland

**DOI:** 10.1186/1897-4287-9-S2-A3

**Published:** 2011-06-01

**Authors:** B Gorski

**Affiliations:** 1 International Hereditary Cancer Center, Pomeranian Medical University, Szczecin, Poland

## 

It is a primary goal in clinical cancer genetics to identify in a population the full range of mutant alleles that predispose to breast cancer (or to another cancer) and then to offer a rapid and inexpensive genetic assay to test for these alleles in a single setting. Many challenges are raised by this approach.. The genetic test must be accurate, rapid and cost effective. If a disease-associated mutation is found, presymptomatic testing of other family members for the specific mutation is possible . In Poland the three founder mutations in BRCA1 (C61G, 4153delA, 5382insC) account for 86% of all BRCA1 and BRCA2 mutations. Mutations in BRCA2 are relatively rare in Poland and no founder BRCA2 mutations have been identified. Several other rare mutations in the BRCA1/2 genes have been found in Polish families with occurrence of hereditary breast and ovarian cancers. Application of Sequenom mutation detection platform, critically improved screening of broad Polish BRCA1/2 genes mutations spectrum. We have developed diagnostic test focused on 25 Polish recurrent BRCA1/2 genes mutations . Mutation detection method was based on automated MALDI–TOF mass spectrometry in Sequenom MassArray™ system using iPLEX GOLD assay reactions that ends after a Single Base Extension.

